# Seasonal PM_2.5_ Exposure and Plasma Metabolome Changes Related to Metabolic Syndrome in Healthy Adults in Chiang Mai, Thailand

**DOI:** 10.3390/toxics14070544

**Published:** 2026-06-23

**Authors:** Puriwat Fakfum, Churdsak Jaikang, Giatgong Konguthaithip, Wason Parklak, Hataichanok Chuljerm, Kanokwan Kulprachakarn

**Affiliations:** 1 School of Health Sciences Research, Research Institute for Health Sciences, Chiang Mai University, Chiang Mai 50200, Thailand; puriwat_f@cmu.ac.th (P.F.); wason.p@cmu.ac.th (W.P.); hataichanok.ch@cmu.ac.th (H.C.); 2 Metabolomic Research Group for Forensic Medicine and Toxicology, Department of Forensic Medicine, Faculty of Medicine, Chiang Mai University, Chiang Mai 50200, Thailand; churdsak.j@cmu.ac.th (C.J.); kongkiat.shang@gmail.com (G.K.)

**Keywords:** air pollution, PM_2.5_ exposure, plasma metabolome, metabolic syndrome, inflammation, oxidative stress

## Abstract

Chiang Mai, Thailand, experiences seasonal fine particulate matter (PM_2.5_) pollution associated with metabolic diseases, but the underlying mechanisms remain unclear. This prospective observational study compared plasma metabolomes of 25 healthy adults in Samoeng District, a highly affected area, between low and high PM_2.5_ exposure seasons using proton nuclear magnetic resonance (^1^H-NMR) spectroscopy. Twenty-six metabolites differentiating haze and non-haze seasons were identified using PLS-DA (VIP > 1.5). During the haze season, 11 were elevated, whereas 15 were decreased. Among the elevated metabolites, the top five—maleylacetoacetic acid, deoxyribose 5-phosphate, betaine, 3-hydroxyanthranilic acid, and 1-methyladenosine—were associated with inflammation, increased reactive oxygen species, nitric oxide inhibition, and altered amino acid metabolism. The top five decreased metabolites—deoxyguanosine, D-arabitol, glycerophosphocholine, ophthalmic acid, and oxaloacetic acid—were involved in several metabolic pathways, particularly those involved in energy metabolism. A total of 56 metabolic pathways were altered by high PM_2.5_ exposure, including pathways related to amino acids, lipids, sugars, nucleotides, vitamins, and energy metabolism. High PM_2.5_ exposure disrupts metabolites and pathways, inducing inflammation, oxidative stress, impaired lipid/energy metabolism, insulin resistance, and high blood pressure. These alterations may increase the risk of metabolic and cardiovascular diseases, with dysregulated metabolites serving as potential biomarkers. These findings highlight the molecular impact of air pollution in affected populations and may support preventive strategies and public health policy development in affected regions. Further studies are needed to clarify these findings.

## 1. Introduction

Air pollution from both atmospheric and indoor environments is a significant global environmental and health risk and is one of the top five leading causes of mortality and morbidity worldwide [1]. The World Health Organization (WHO) estimates that more than 23% of global deaths are associated with environmental factors. Of the 12.6 million deaths caused by non-communicable diseases (NCDs) each year, approximately two-thirds result from exposure to environmental risk factors. Therefore, environmental risk factors and NCDs pose a significant threat to global public health [2]. Fine particulate matter or PM_2.5_ is defined as particles smaller than 2.5 micrometers in diameter that can cause health effects. These particles are present in the atmosphere and are emitted from various sources, such as forest fires and human activities, including fuel burning and industrial processes [3]. PM_2.5_ in the air has become a significant global health concern, particularly in developing countries. Numerous studies report that PM_2.5_ is a significant risk factor for human health and mortality [4,5]. Exposure to ambient PM_2.5_ has been shown to be associated with an increased incidence and mortality of non-communicable diseases [6], as well as a higher risk of mortality from diabetes [7]. Exposure to PM_2.5_ has also been linked to the pathogenesis of chronic diseases associated with metabolic syndrome, including cardiovascular diseases [8] and diabetes [9]. The biological mechanisms underlying the pathogenesis of metabolic diseases are associated with oxidative stress, inflammatory responses, and genotoxicity [10].

Samoeng District, Chiang Mai, Thailand, is a region severely affected by wildfires, leading to significantly high levels of PM_2.5_ pollution during the haze season, exceeding standard limits. According to the Geo-Informatics and Space Technology Development Agency (GISTDA), the average PM_2.5_ level during the haze season in Samoeng District, particularly during agricultural burning, is typically three to five times higher than the WHO standard. Moreover, PM_2.5_ concentrations in Samoeng District in March 2019 exceeded 500 μg/m^3^, indicating a very high level of air pollution [11]. Studies on the health impacts of PM_2.5_ exposure among residents of Samoeng District have found that exposure is associated with upregulation of inflammatory markers, including interleukin-6 (IL-6), especially among individuals with metabolic syndrome [12], as well as elevated oxidative stress biomarkers such as malondialdehyde (MDA), 8-epi-prostaglandin F_2_α (8-epi-PGF2α), and 1-hydroxypyrene (1-OHP) during the season of high PM_2.5_ exposure [13]. However, the relationship between epidemiological evidence and pathogenic mechanisms in metabolic diseases needs to be clearly established through the analysis of metabolic changes in related genes and proteins. Particularly, metabolomics is a powerful tool that can be used to identify and quantify metabolites that reflect the biochemical processes underlying various physiological conditions and characterization of complex phenotypes [14,15]. There have been studies on metabolic profile changes associated with PM_2.5_ exposure, especially in high-exposure areas in Chiang Mai, Thailand. Jaikang et al. conducted a study on the effects of long-term PM_2.5_ exposure on the blood metabolome of individuals from areas with low and high levels of PM_2.5_ using ^1^H-NMR-based techniques [16,17]. NMR spectroscopy provides several advantages, including high reproducibility and the ability to identify unknown metabolites in complex biological samples. It is also highly automatable and supports high-throughput analyses, while requiring minimal or no sample preparation or chemical derivatization [18]. Metabolomics results show that exposure to PM_2.5_ was involved in impairments in energy and amino acid metabolism and contributed to increased oxidative stress and inflammation, particularly through disruption of the kynurenine pathway. These metabolic disturbances caused by air pollution are linked to an increased risk of NCDs [16,17]. However, there is limited evidence comparing the blood metabolome of individuals exposed to high and low levels of PM_2.5_ during the haze and non-haze seasons, especially in Chiang Mai Province, Thailand, which consistently experiences high levels of air pollution.

Therefore, this study aims to evaluate the impact of PM_2.5_ exposure on metabolic variations and pathway disruptions in the plasma metabolome through ^1^H-NMR-based analyses. By comparing individuals exposed to high and low levels of PM_2.5_ during the haze and non-haze seasons in Samoeng District, Chiang Mai Province, Thailand, this study aims to elucidate metabolomic changes that may explain the mechanisms by which PM_2.5_ exposure during the haze season contributes to metabolic diseases and help identify potential biomarkers involved in disease pathogenesis.

## 2. Materials and Methods

### 2.1. Study Population

Healthy participants, both male and female (not breastfeeding or not pregnant), were recruited from Samoeng District, Chiang Mai Province, Thailand. The inclusion criteria were: (1) age between 20 and 60 years; (2) residence in the Samoeng District for at least five years; and (3) no history of serious diseases, including chronic kidney disease, cardiovascular diseases, cancer, or presence of active infections during the enrollment period. Baseline information, including demographic characteristics, socioeconomic status, and lifestyle data, was collected.

### 2.2. Study Area and Air Quality Monitoring

Samoeng District in Chiang Mai Province was selected for blood sample collection from volunteers due to its unique environment and geography. Air quality monitoring in this area has been described in detail previously [17]. This district experiences high levels of PM_2.5_ during the haze season, largely because its valley-like geography allows pollutants and smoke from wildfires and other sources to accumulate. The haze season was defined as March–April 2023, whereas the non-haze season was defined as May–July 2023. Daily PM_2.5_ concentrations were obtained from the Northern Thailand Air Quality Health Index (NTAQHI) database, based on data from air quality monitoring stations in Samoeng district. 

### 2.3. Study Design, Sample Collection and Ethical Approval

Ethical approval for this study was granted by the Human Experimentation Committee of the Research Institute for Health Sciences, Chiang Mai University (Approval No. 03/2023) on 19 January 2023. Written informed consent was obtained from all participants before enrollment. Demographic data, physical examination measurements, vital signs, and plasma samples were collected from healthy individuals residing in Samoeng District, Chiang Mai, Thailand, all of whom had lived in the district for at least five years to ensure consistency in environmental exposure. Sampling was conducted across two seasons: the haze season (high PM_2.5_ exposure) and the non-haze season (low PM_2.5_ exposure). Blood samples were collected from 25 healthy individuals after an overnight fast in late April 2023 (end of the haze season) and late July 2023 (end of the non-haze season) for laboratory measurements. Plasma was separated using heparinized tubes followed by centrifugation at 3000× *g* for 10 min and used for metabolomic analysis.

### 2.4. Preparation of Plasma Samples

All plasma samples were stored at −80 °C until analysis, and repeated freeze–thaw cycles were avoided. Plasma samples were analyzed using Nuclear Magnetic Resonance (NMR) spectroscopy following the protocol described by Jaikang et al. [16]. Briefly, each plasma sample was lyophilized and subsequently mixed with 0.6 mL of 0.1 M trimethylsilyl propanoic acid (TSP) in deuterium oxide (D_2_O) as internal standard, then transferred into an NMR tube. NMR measurements at 500 MHz were performed with water suppression to reduce the intensity signal of water. All reagents were obtained from the same batch, and all samples were analyzed under consistent experimental conditions.

### 2.5. Acquisition of ^1^H-NMR Spectra

Acquisition of ^1^H-NMR spectra was performed following the protocol described by Jaikang et al. [17]. Briefly, ^1^H-NMR spectra of human plasma were acquired using a Bruker AVANCE 500 MHz spectrometer (Bruker BioSpin, Rheinstetten, Germany), equipped with a Carr–Purcell–Meiboom–Gill (CPMG, RD–90^−^(τ–180^−^τ)^n^–acquire) pulse sequence (Bruker BioSpin, Rheinstetten, Germany). The spectra were captured at 27 °C. The NMR acquisition parameters were a dwell time (DW) of 60.40 µs, a free induction decay (FID) resolution of 0.126 Hz, a spectral width of 8278.146 Hz, a relaxation delay of 1 s, 16 scans, and an acquisition time of 3.95 s. A 90° pulse with sixteen signal averages (NSAs) was applied. TopSpin 4.0.7 software was used for processing the NMR spectra, including baseline and phase correction. Spectra in the range of 0 to 12 ppm were analyzed, with the data normalized to the total integrated area.

### 2.6. Metabolite Identification by Database and Spectral Analysis

Metabolites were identified using the Human Metabolome Database (HMDB) by matching the ^1^H-NMR chemical shifts with those reported in the database. For spectral analysis, J-coupling analysis, chemical shift and spin–spin coupling patterns were performed. Each metabolite was identified with high accuracy by confirming that its chemical shift matched the HMDB reference values within a deviation of 0.01 ppm [16,18].

### 2.7. Data Processing and Analysis

MestReNova software (version 12.0.0; Mestrelab Research, Santiago de Compostela, Spain) was utilized for the processing, analysis, and interpretation of the ^1^H-NMR spectra. Metabolite concentrations were quantified based on signal intensities using the following equation.

$$\frac{I_{A}}{I_{B}}=\frac{H_{A}}{H_{B}}\times \frac{C_{A}}{C_{B}}$$

where: $I_{A}$ = Signal intensity of the metabolite, $I_{B}$ = Signal intensity of TSP, $H_{A}$ = Number of protons in the metabolite, $H_{B}$ = Number of protons in TSP, $C_{A}$ = Metabolite concentration, $C_{B}$ = TSP concentration (µM).

### 2.8. Statistical Analysis

Descriptive statistics were used to summarize the baseline characteristics of all subjects. Differences in physical and laboratory characteristics—including body weight and height, body mass index (BMI), hip and waist circumference, blood pressure, heart rate, blood glucose, lipid profiles, serum creatinine, and blood urea nitrogen (BUN)—between the non-haze and haze seasons within the same healthy individuals were analyzed using the paired *t*-test in SPSS software, version 15.0 (SPSS Inc., Chicago, IL, USA). A *p*-value < 0.05 was considered statistically significant.

### 2.9. Metabolomics Data Analysis

MetaboAnalyst version 6.0 (http://www.metaboanalyst.ca/MetaboAnalyst, accessed on 12 January 2025) is a free online platform for the processing, analysis, and interpretation of metabolomics data. Non-negative values were verified and missing values were eliminated before analysis. Median, log transformation (logbase2), and auto-scaling were used for data normalization. Partial least squares discriminant analysis (PLS-DA) was employed to investigate differences in metabolites of healthy individuals between the haze season (high PM_2.5_ exposure) and the non-haze season (low PM_2.5_ exposure). The Variable Importance for the Projection (VIP) scores calculated from the PLS-DA model in MetaboAnalyst 6.0 were used to identify important variables and determine discriminatory metabolites in the dataset. The VIP scores ≥ 1.5 were considered significant discriminatory metabolites. Detailed information about these metabolites was obtained from the Human Metabolome Database (HMDB version 5.0).

## 3. Results

### 3.1. Participant Demographic Information

Table 1 presents the baseline characteristics of 25 healthy individuals, including age, gender, smoking status, alcohol consumption, educational level, and income. The average age of the participants was 48.3 ± 14.3 years. There were more females (60.0%) than males (40.0%). Most participants were non-smokers (72.0%), and 76.0% reported alcohol consumption. Most participants had at least primary education, and the largest group had completed senior high school (44.0%). In terms of income, most participants were in the middle-income group (44.0%). Physical and laboratory characteristics between the non-haze and haze seasons within the same individuals showed no statistically significant differences, as summarized in Table 2.

### 3.2. PM_2.5_ Concentrations During Haze and Non-Haze Seasons

Using data from the Northern Thailand Air Quality Health Index (NTAQHI), the mean daily PM_2.5_ concentration during the haze season (March–April 2023) was 77.78 ± 47.33 µg/m^3^, whereas during the non-haze season (May–July 2023), the mean concentration was 8.36 ± 7.50 µg/m^3^.

### 3.3. Analysis of Differentially Expressed Metabolites of Healthy Individuals Between the Haze Season (High PM2.5 Exposure) and the Non-Haze Season (Low PM2.5 Exposure)

**Figure 1:**
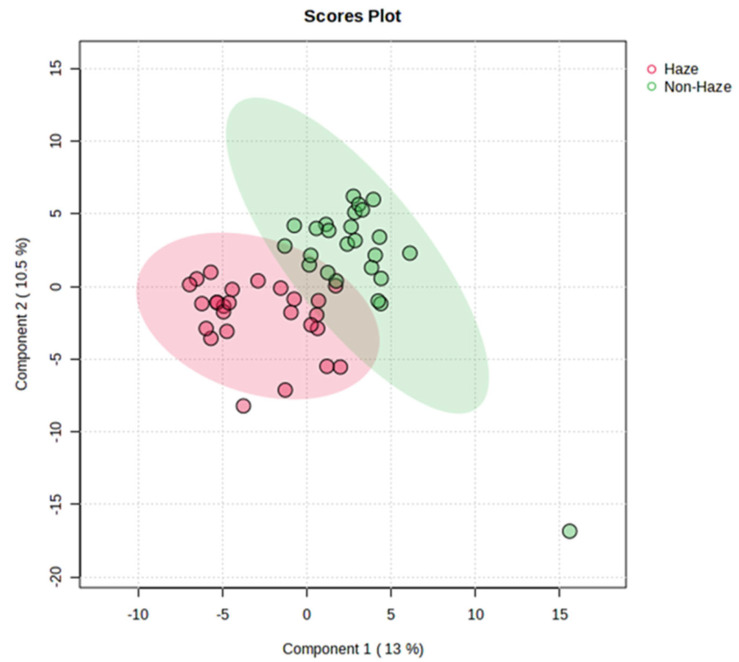
Differences in plasma metabolites of healthy individuals between haze and non-haze seasons using the partial least squares discriminant analysis (PLS-DA) plot. Component 1 (13%) and Component 2 (10.5%) explain the variation in the dataset.

In this study, ^1^H-NMR spectroscopy was used to identify 209 metabolites in blood samples of 25 healthy individuals between the haze season (high PM_2.5_ exposure) and the non-haze season (low PM_2.5_ exposure). The differences in blood metabolites of healthy individuals between the two seasons (haze and non-haze) are shown according to PLS-DA plots in Figure 1. The PLS-DA plot demonstrates a clear separation between seasons, with components 1 and 2 accounting for 13% and 10.5%, respectively.

**Figure 2:**
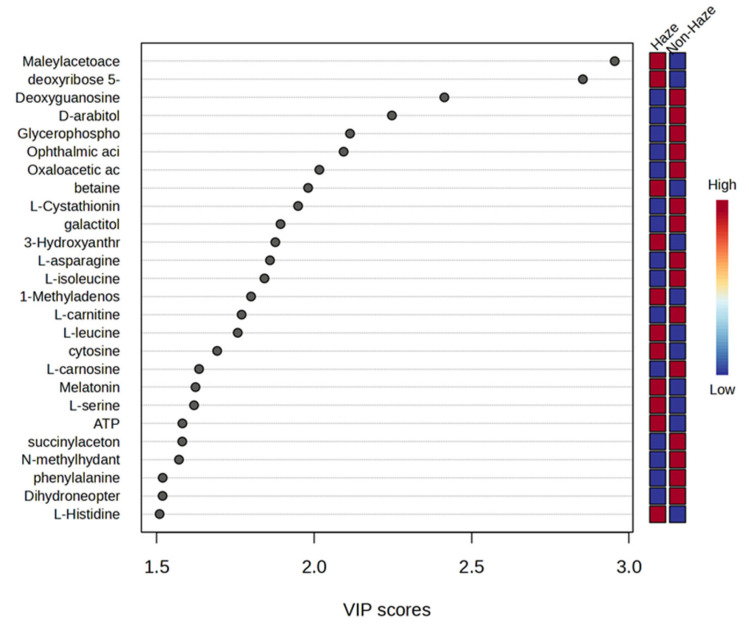
The 26 key metabolites differentiating haze and non-haze seasons in healthy individuals, identified using the MetaboAnalyst PLS-DA module with VIP scores. The grey dots represent the VIP score values for each metabolite. The colored boxes on the right show metabolite levels, where red means higher levels and blue means lower levels in each season. Full metabolite names are provided in Supplementary Table S1.

Twenty-six key metabolites differentiating haze and non-haze periods in healthy individuals were identified using the MetaboAnalyst PLS-DA module with VIP > 1.5, as shown in Figure 2 and Supplementary Table S1. Eleven metabolites were markedly elevated, while 15 metabolites were markedly lower during the haze season (high PM_2.5_ exposure) compared with the non-haze season (low PM_2.5_ exposure). Among the elevated metabolites, the top five were maleylacetoacetic acid, deoxyribose 5-phosphate, betaine, 3-hydroxyanthranilic acid, and 1-methyladenosine, which are associated with inflammation, increased reactive oxygen species (ROS) levels, nitric oxide (NO) inhibition, and amino acid metabolism. Conversely, the top five lowered metabolites including deoxyguanosine, D-arabitol, glycerophosphocholine, ophthalmic acid, and oxaloacetic acid are involved in metabolic processes, for instance, purine metabolism, the pentose phosphate pathway, choline metabolism, glutamine derivatives, and the TCA cycle. The reduction in these metabolites leads to disruptions in metabolic pathways, particularly those involved in energy metabolism.

### 3.4. Pathway Impact Analysis in Healthy Individuals Exposed to High and Low Levels of PM_2.5_

**Figure 3:**
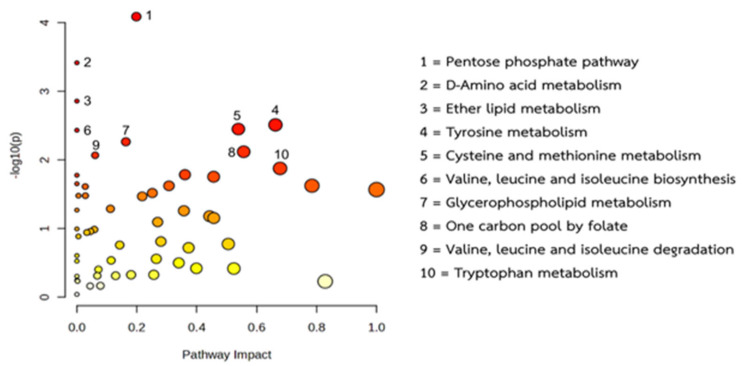
Pathway analysis of healthy individuals exposed to high and low levels of PM_2.5_. Darker red dots indicate higher statistical significance, and the size of the circles represents the pathway impact values. The *X*-axis represents pathway impact.

The relevant pathways that match based on effect values from topology analysis and *p*-values from pathway analysis are presented in Figure 3. A total of 56 pathways were altered by PM_2.5_ exposure, including those involved in the metabolism of amino acids, lipids, carbohydrates, nucleotides, vitamins and cofactors, as well as energy metabolism. The pathway impacts in healthy individuals exposed to high and low levels of PM_2.5_ during the haze and non-haze seasons are shown in Table 3. Notably, the pentose phosphate pathway was identified as the most dysregulated pathway, exhibiting the highest statistical significance. Meanwhile, several pathways showed high pathway impacts, including tyrosine metabolism and cysteine and methionine metabolism. In contrast, some pathways showed moderate impact with lower statistical significance, suggesting different levels of metabolic changes across pathways.

## 4. Discussion

This prospective observational study examined changes in the plasma metabolome during the haze season. Our findings indicate that PM_2.5_ exposure is associated with alterations in both plasma metabolites and metabolic pathways. The results also showed that PM_2.5_ exposure during the haze season was associated with notable alterations in plasma metabolites, including increased levels of metabolites related to inflammation, nitrative stress, oxidative stress, elevated ROS, and amino acid metabolism. In addition, lower levels of certain metabolites indicated possible disruptions in metabolic processes, including cellular energy metabolism and nucleic acid metabolism.

Notably, maleylacetoacetic acid was the most upregulated metabolite under PM_2.5_ exposure during the haze season. This metabolite is involved in tyrosine degradation and has been linked to increased reactive nitrogen species (RNS) production and nitrative stress, which may contribute to inflammatory processes [19,20]. These observations are consistent with previous reports showing that environmental and air pollution exposures can promote nitrative stress and cause damage to various biomolecules [21]. However, metabolomic evidence linking maleylacetoacetic acid to PM_2.5_ exposure remains limited. Therefore, our findings suggest that its increase may reflect PM_2.5_-induced inflammation through nitrative stress.

Interestingly, levels of glycerophosphocholine, ophthalmic acid, oxaloacetic acid, L-cystathionine, galactitol, L-asparagine, and L-isoleucine were markedly decreased due to high PM_2.5_ exposure. Glycerophosphocholine is a key metabolite involved in reducing ROS and promoting longevity in humans [22]. Ophthalmic acid and oxaloacetic acid are associated with blood glucose regulation. Increased levels of ophthalmic acid have been linked to enhanced glucose uptake in experimental models, while oxaloacetic acid is involved in metabolic pathways related to improved insulin sensitivity and reduced blood glucose levels in human studies, as well as lifespan extension [23]. L-cystathionine has been reported to exert protective effects against oxidative stress-related cardiovascular diseases by reducing oxidative damage and preventing subsequent DNA damage induced by oxidized low-density lipoprotein [24]. L-asparagine has been associated with stimulating insulin release [25]. L-isoleucine plays a role in energy metabolism and protein synthesis and has been reported to enhance muscle glucose uptake and prevent tissue triglyceride accumulation in animal studies [26,27,28]. These findings indicate that reduced levels of these metabolites under high PM_2.5_ exposure during the haze season may be linked to oxidative stress, impaired glucose uptake, and DNA damage, contributing to an increased risk of cardiovascular disease and accelerated aging.

Additionally, our results show that high PM_2.5_ exposure during the haze season up-regulated the levels of betaine, 3-hydroxyanthranilic acid, 1-methyladenosine, L-leucine, and ATP. Human studies have reported that increased plasma betaine levels are associated with pulmonary hypertension (PH), CVDs, and diabetes, as well as disease severity, while elevated levels of betaine and 3-hydroxyanthranilic acid have been observed in individuals with type 2 diabetes mellitus (T2DM) [29,30,31,32]. Previous studies have linked 1-methyladenosine levels to various diseases, including gestational diabetes in humans and hypertension. In addition, increased levels of 1-methyladenosine in the blood of mice have been linked to hypertension through the activation of inflammasomes [33]. L-leucine, a branched-chain amino acid (BCAA), has been implicated in several pathological conditions. Elevated blood L-leucine is associated with obesity, insulin resistance, T2DM, and a higher risk of coronary artery disease. Importantly, elevated levels of BCAAs, including L-leucine, can lead to endothelial dysfunction by triggering inflammation and oxidative stress [34]. Therefore, the increased levels of these metabolites with high PM_2.5_ exposure during the haze season indicate a potential dysregulation of key metabolic pathways, which may contribute to the development of metabolic syndrome-related conditions. In addition, elevated melatonin levels may indicate a protective response to oxidative stress. Consistently, increased urinary melatonin in humans has been associated with air pollution–induced oxidative stress and may act as a protective mechanism against its adverse effects [35]. These results suggest that high PM_2.5_ exposure during the haze season triggers oxidative stress. However, we observed higher plasma L-serine levels, whereas a previous study reported that down-regulation of serine was associated with air pollution-induced arterial stiffening [36]. Further studies are needed to clarify the role of L-serine in response to PM_2.5_ exposure during the haze season. Moreover, high PM_2.5_ exposure during the haze season was associated with increased plasma ATP levels. Previous studies have linked elevated ATP levels to higher blood pressure in hypertensive patients [37]. Consistently, our findings showed a slight increase in both systolic and diastolic blood pressure during the haze season (*p* = 0.056 and 0.057, respectively), suggesting a potential association between PM_2.5_ exposure and elevated blood pressure risk.

Importantly, we observed lower levels of L-carnitine and L-carnosine associated with high PM_2.5_ exposure during the haze season. L-carnitine plays an important role in long-chain fatty acid transport, fatty acid oxidation, and ATP production, while both metabolites are associated with cardioprotective effects through reducing oxidative stress, inflammation, and metabolic risk factors [38,39,40,41]. Higher plasma L-carnitine levels have also been linked to a lower risk of CVDs [39]. These findings suggest that reduced levels of these metabolites may contribute to the progression of metabolic diseases, CVDs, and age-related conditions.

To further investigate the biological impacts of PM_2.5_ exposure during the haze season, we performed pathway analysis using MetaboAnalyst 6.0. Pathway impact analysis revealed that high PM_2.5_ exposure dysregulated 56 metabolic pathways, most of which were associated with amino acid metabolism, followed by pathways involved in lipid, carbohydrate, and purine metabolism. We found that the pentose phosphate pathway was the most dysregulated pathway under high PM_2.5_ exposure during the haze season. An animal study has shown that PM_2.5_ exposure alters the pentose phosphate pathway, leading to increased fatty acid synthesis, which is associated with insulin resistance [42]. Additionally, high PM_2.5_ exposure during the haze season disturbed tyrosine metabolism. A metabolomic study of young adults in China found that long-term PM_2.5_exposure could disrupt tyrosine metabolism. Disorders in tyrosine metabolism may lead to the accumulation of ROS, thereby inducing oxidative stress [43]. In this study, high PM_2.5_ exposure during the haze season also interfered with cysteine, methionine, alanine, aspartate, glutamate, serine, threonine, and glycine metabolism, as well as valine, leucine, and isoleucine biosynthesis. A human study reported that these metabolic pathways were associated with the toxic metabolic effects of PM_2.5_ in the liver [44]. We also found that PM_2.5_ disrupted sphingolipid metabolism. According to a prospective panel study in a Chinese population, dysregulation of sphingolipid metabolism is associated with the development of atherosclerosis following PM_2.5_ exposure [45]. The disturbances in metabolic pathways and the altered metabolite levels observed in our study are consistent with each other, both of which are associated with the development of metabolic syndrome and its related diseases, including CVDs.

Taken together, the results show that high PM_2.5_ exposure during the haze season increases several metabolites that are associated with increased oxidative stress, inflammation, insulin resistance, elevated blood pressure, and obesity—processes that play crucial roles in the progression of metabolic syndrome-related conditions. Moreover, higher levels of some metabolites may be linked to an increased risk of metabolic syndrome and CVDs. In contrast, the metabolites that decreased following PM_2.5_ exposure during the haze season are key regulators involved in reducing oxidative stress, inflammation, blood glucose, blood pressure, and DNA damage, as well as promoting longevity and improving hyperlipidemia and energy metabolism. Together with pathway analysis, we also found that PM_2.5_-induced disturbances in metabolic pathways are associated with ROS generation, insulin resistance, and the development of atherosclerosis. These findings align with previous reports indicating that PM_2.5_ induces inflammation, oxidative stress, and endothelial dysfunction, ultimately leading to obesity, lipid metabolism disorders, hypertension, hyperglycemia, and DNA damage [46]. Overall, our findings indicate that high PM_2.5_ exposure during the haze season may increase the risk of metabolic syndrome-related conditions through metabolite alterations and dysregulation of metabolic pathways involved in biological mechanisms such as inflammation, oxidative stress, and abnormal lipid and energy metabolism, together with dysregulated levels of metabolite biomarkers associated with CVDs and related diseases.

Our study has certain limitations that future research should address. First, it is a preliminary investigation with a relatively small sample size, which was determined based on the sample sizes used in previous studies. A small proportion of participants were active smokers (16.0%), which may act as a potential confounder. However, the paired design, where each participant served as their own control, helped reduce this effect. Second, we did not investigate other potential confounding factors that may affect changes in metabolite levels and metabolic pathways. Finally, the lack of data on the chemical characterization of PM_2.5_ limits our ability to understand its potential effects on metabolite pathways and changes in metabolite levels. Further studies are needed to increase the sample size and expand the study areas. In addition, a longitudinal design and air sampling for PM_2.5_ analysis are needed to better understand the health impacts of PM_2.5_ exposure. Future research should also investigate confounding factors and examine the relationship between the chemical composition of PM_2.5_ and changes in metabolite levels and metabolic pathways to improve the accuracy of our findings.

However, our study also has several strengths. First, this is the first paired-case, prospective observational study in Thailand conducted among participants living in areas with high PM_2.5_ levels during the haze season and in a community with valley-like geography that allows wildfire smoke to accumulate. Second, our findings revealed changes in many metabolites that may be linked to biological mechanisms associated with the development of various metabolic conditions and related diseases induced by high PM_2.5_ exposure during the haze season. These findings highlight the potential molecular impact of air pollution exposure in local populations. They provide supporting evidence for public health policies and mitigation strategies in Chiang Mai, where seasonal air pollution continues to be a significant concern.

## 5. Conclusions

In conclusion, the results suggest that high PM_2.5_ exposure during the haze season was associated with disruptions in key metabolites and metabolic pathways, including those related to inflammation, oxidative stress, and lipid and energy metabolism. These alterations may contribute to potential metabolic dysfunctions, such as insulin resistance and elevated blood pressure, and may be linked to an increased potential risk of metabolic syndrome and cardiovascular diseases. In addition, high PM_2.5_ exposure during the haze season was associated with dysregulated metabolite levels that may serve as potential biomarkers for CVDs and related conditions. Our findings should inform preventive strategies and guide public health policies in Thailand and neighboring regions across Southeast Asia, where seasonal haze continues to affect large populations, emphasizing the importance of increased public awareness and effective regional mitigation strategies. However, further research with larger sample sizes and a longitudinal design are essential to clarify the impacts of high PM_2.5_ during haze season exposure in affected regions and populations.

## Figures and Tables

**Table 1:** Baseline demographic characteristics of the study population (*n* = 25).

Characteristic	Healthy Individuals (*n* = 25)
Age, Year, Mean ± SD	48.3 ± 14.3
Gender, *n* (%)	
Male	10 (40.0)
Female	15 (60.0)
Smoking status, *n* (%)	
Never	18 (72.0)
Current	4 (16.0)
Former	3 (12.0)
Alcohol consumption, *n* (%)	
Yes	19 (76.0)
No	6 (24.0)
Level of education, *n* (%)	
Illiterate	1 (4.0)
Lower primary education	2 (8.0)
Upper primary education	8 (32.0)
Junior high school	3 (12.5)
Senior high school	11 (44.0)
Income, *n* (%)	
Less than 5000 Thai Baht	4 (16.0)
5001–15,000 Thai Baht	11 (44.0)
15,001–25,000 Thai Baht	9 (36.0)
More than 25,001 Thai Baht	1 (4.0)

**Table 2:** Physical and laboratory characteristics of healthy individuals exposed to high and low levels of PM_2.5_ during haze and non-haze seasons, with statistical analysis.

Characteristic	Low Levels of PM_2.5_ (Non-Haze Season, *n* = 25)	High Levels of PM_2.5_ (Haze Season, *n* = 25)	*p*-Value
Body weight (kg)	60.0 ± 10.3	59.4 ± 14.4	0.555
Body height (cm)	157.9 ± 7.6	156.2 ± 7.6	0.390
Body mass index (kg/m^2^)	24.0 ± 3.4	24.3 ± 5.0	0.747
Hip Circumference (cm)	94.0 ± 7.6	93.6 ± 10.5	0.509
Waist circumference (cm)	79.9 ± 8.9	79.9 ± 13.7	0.575
Systolic BP (mmHg)	125.6 ± 19.6	134.6 ± 19.8	0.056
Diastolic BP (mmHg)	78.2 ± 10.2	83.8 ± 10.6	0.057
Heart rate (bpm)	71.0 ± 8.4	74.0 ± 9.3	0.249
Fasting blood glucose (mg/dL)	86.3 ± 11.7	83.7 ± 7.2	0.612
Triglyceride (mg/dL)	126.1 ± 42.7	133.0 ± 78.5	0.640
Total cholesterol (mg/dL)	206.9 ± 40.8	206.8 ± 29.8	0.921
LDL-cholesterol (mg/dL)	113.1 ± 33.5	122.7 ± 32.0	0.283
HDL-cholesterol (mg/dL)	55.7 ± 13.0	55.2 ± 11.1	0.848
Blood urea nitrogen (mg/dL)	14.5 ± 3.5	13.5 ± 3.6	0.217
Serum creatinine (mg/dL)	0.9 ± 0.2	0.8 ± 0.2	0.143

Note: *p*-value represents the statistical comparison between the non-haze and haze seasons within the same healthy individuals. Data are presented as mean ± standard deviation (SD).

**Table 3:** Comparative pathway impact analysis in healthy individuals exposed to high and low levels of PM_2.5_ during the haze and non-haze seasons.

Pathways	Match Status	−log(p)	Impact
Pentose phosphate pathway	2/23	4.0871	0.19845
D-Amino acid metabolism	2/15	3.4153	0.0
Ether lipid metabolism	1/20	2.8575	0.0
Tyrosine metabolism	12/42	2.5113	0.66262
Cysteine and methionine metabolism	8/33	2.45	0.53894
Valine, leucine and isoleucine biosynthesis	6/8	2.433	0.0
Glycerophospholipid metabolism	5/36	2.265	0.16336
One carbon pool by folate	13/26	2.1196	0.55661
Valine, leucine and isoleucine degradation	8/40	2.0681	0.06088
Tryptophan metabolism	12/41	1.8771	0.67802
Histidine metabolism	6/16	1.7857	0.36065
Sphingolipid metabolism	1/32	1.7775	0.0
Glycine, serine and threonine metabolism	11/33	1.7554	0.45638
Galactose metabolism	4/27	1.6529	0.0
Alanine, aspartate and glutamate metabolism	13/28	1.6248	0.78446
Purine metabolism	15/70	1.6237	0.3073
Porphyrin metabolism	2/31	1.6125	0.02795
Phenylalanine, tyrosine and tryptophan biosynthesis	3/4	1.5685	1.0
Glyoxylate and dicarboxylate metabolism	12/32	1.5185	0.25167
Steroid biosynthesis	1/41	1.4792	0.02837

## Data Availability

The original contributions presented in this study are included in the article/Supplementary Materials. Further inquiries can be directed to the corresponding author.

## Supplementary Materials

The following supporting information can be downloaded at: https://www.mdpi.com/article/10.3390/toxics14070544/s1, Table S1: Full metabolite annotations and VIP scores for the discriminatory metabolites identified by Partial Least Squares Discriminant Analysis (PLS-DA).

## Abbreviations

The following abbreviations are used in this manuscript:

ATP	Adenosine triphosphate
BCAAs	Branched-chain amino acids
BMI	Body mass index
BUN	Blood urea nitrogen
CPMG	Carr–Purcell–Meiboom–Gill
CVDs	Cardiovascular diseases
D_2_O	Deuterium oxide
DW	Dwell time
FID	Free induction decay
GISTDA	Geo-Informatics and Space Technology Development Agency
HMDB	Human Metabolome Database
IL-6	Interleukin-6
MDA	Malondialdehyde
NCDs	Non-communicable diseases
NMR	Nuclear magnetic resonance
NO	Nitric oxide
NSA	Number of signal averages
NTAQHI	Northern Thailand Air Quality Health Index
PH	Pulmonary hypertension
PLS-DA	Partial least squares discriminant analysis
PM_2.5_	Particulate matter with aerodynamic diameter ≤ 2.5 µm
RNS	Reactive nitrogen species
ROS	Reactive oxygen species
SD	Standard deviation
SPSS	Statistical Package for the Social Sciences
T2DM	Type 2 diabetes mellitus
TCA	Tricarboxylic acid cycle
TSP	Trimethylsilyl propanoic acid
VIP	Variable importance in projection
WHO	World Health Organization
^1^H-NMR	Proton nuclear magnetic resonance
8-epi-PGF_2_α	8-epi-prostaglandin F2 alpha
1-OHP	1-Hydroxypyrene
