# Supplementary material for: Seasonal PM2.5 Exposure and Plasma Metabolome Changes Related to Metabolic Syndrome in Healthy Adults in Chiang Mai, Thailand
Source: Toxics. 2026 Jun 23;14(7):544. doi: 10.3390/toxics14070544 (PMC13418832; doi:10.3390/toxics14070544)
Supplement: Supplementary file 1 [file toxics-14-00544-s001.zip › toxics-4349852-supplementary.pdf]

Supplementary Materials

Supplementary Table S1. Full metabolite annotations and VIP scores for the discriminatory metabolites identified by Partial Least Squares Discriminant Analysis (PLS-DA).

No.	Metabolite (short name in Fig. 2)	Full Metabolite Name	VIP scores
1	Maleyacetoace	Maleylacetoacetic acid	2.9547
2	deoxyribose 5-	Deoxyribose 5-phosphate	2.8535
3	Deoxyguanosine	Deoxyguanosine	2.4136
4	D-arabitol	D-arabitol	2.2471
5	Glycerophospho	Glycerophosphocholine	2.1139
6	Ophthalmic aci	Ophthalmic acid	2.0937
7	Oxaloacetic ac	Oxaloacetic acid	2.0166
8	betaine	Betaine	1.9809
9	L-Cystathionin	L-Cystathionine	1.9491
10	galactitol	Galactitol	1.8933
11	3-Hydroxyanthr	3-Hydroxyanthranilic acid	1.8768
12	L-asparagine	L-asparagine	1.8598
13	L-isoleucine	L-isoleucine	1.8423
14	1-Methyladenos	1-Methyladenosine	1.7997
15	L-carnitine	L-carnitine	1.7697
16	L-leucine	L-leucine	1.7574
17	cytosine	Cytosine	1.692
18	L-carnosine	L-carnosine	1.6346
19	Melatonin	Melatonin	1.6232
20	L-serine	L-serine	1.6187
21	ATP	ATP	1.5817
22	succinylaceton	Succinylacetone	1.5812
23	N-methylhydant	N-methylhydantoin	1.5707

24	phenylalanine	Phenylalanine	1.519
25	Dihydroneopter	Dihydroneopterin triphosphate	1.5187
26	L-Histidine	L-Histidine	1.5092
